# Papillary thyroid carcinoma presenting as a primary renal tumor with multiple pulmonary and bone metastases: a case report

**DOI:** 10.1186/s13256-019-2025-8

**Published:** 2019-04-20

**Authors:** Emre Gezer, Alev Selek, İlhan Tarkun, Zeynep Cantürk, Berrin Çetinarslan

**Affiliations:** Department of Endocrinology and Metabolism, Faculty of Medicine, Kocaeli University, Kocaeli, Turkey

**Keywords:** Papillary thyroid carcinoma, Renal tumor, Distant metastasis

## Abstract

**Background:**

Papillary thyroid carcinoma is the most common endocrine malignancy. Distant metastasis from differentiated thyroid carcinoma is infrequent and the metastasis rate of papillary thyroid carcinoma is lower than that of follicular thyroid carcinoma. Distant metastases from differentiated thyroid carcinoma are usually seen in the lungs and bones; however, renal metastasis is very rare.

**Case presentation:**

Here we describe an 85-year-old Caucasian woman who presented with right flank pain 10 years ago. We describe a case of papillary thyroid carcinoma presenting as a primary renal tumor with extensive pulmonary and bone metastases. Abdominal screening with computed tomography revealed a mass on her right kidney, which was considered a primary renal cell carcinoma and she underwent a right nephrectomy. Unexpectedly, papillary thyroid carcinoma metastasis was diagnosed from demonstrative histopathological findings, such as positive immunoperoxidase staining for thyroglobulin. A total thyroidectomy was performed. Unenhanced thoracic computed tomography and skeletal scintigraphy revealed bilateral multiple nodules in her lungs and bone metastasis on T10 vertebra and right sacroiliac joint. Initially, 30 Gy radiotherapy was implemented to her T9–10 vertebrae and then she was treated with a total of 800 mCi radioactive iodine for ablation. A radioactive iodine whole body scan was performed after each 200 mCi and continuous progression was shown in each scan. After she was lost to follow-up for 3 years, she referred to our clinic again with a draining mass on her neck and we planned radiotherapy to this giant mass.

**Conclusion:**

Our patient was surprisingly still alive after metastatic disease was diagnosed 10 years ago and she had no major complaint other than a draining mass on her neck. Our primary aim by sharing this case is to underline potential renal metastasis from papillary thyroid carcinoma. In other words, when approaching primary renal tumors, possible distant metastases of other organs need to be kept in mind for differential diagnosis. In addition, it should be noted that if managed appropriately, the long-term survival in patients with papillary thyroid carcinoma with multiple organ metastases could be encouraging.

## Background

Papillary thyroid carcinoma (PTC) is the most common thyroid malignancy, accounting for 85% of all thyroid malignancies. PTC originates from thyroid follicular epithelial cells. In contrast to this high incidence, distant metastases of PTC are rarely seen, whereas metastases of follicular thyroid carcinoma (FTC) are more frequent, probably due to its vascular invasion [1]. Distant metastases of differentiated thyroid carcinoma (DTC) are usually seen at lungs and bones, whereas renal metastasis is very rare. Moreover, metastatic PTC presenting as a primary renal tumor is especially uncommon, as case reports of patients with metastatic FTC are more frequent in the literature [2, 3]. As expected, there are low nominal survival rates of patients with DTC with distant metastases, as in other cancers.

Here we present a case of PTC presenting as a primary renal tumor with extensive pulmonary and bone metastases. Our primary aims by reporting this case are to emphasize the importance of approaching renal tumors from a different point of view and being aware of the possible good prognosis of even an aggressive metastatic PTC, considering radioactive iodine (RAI) as the most efficient treatment for PTCs with distant metastases.

## Case presentation

An 85-year-old Caucasian woman presented to our hospital with right flank pain 10 years ago. She had a past medical history of type 2 diabetes mellitus and essential hypertension. She denied any history of thyroid disease and neck irradiation. She had no family history of any cancer. She was a housewife and had no history of tobacco smoking or consuming alcohol. A physical examination at the time of presentation was not significant except for right costovertebral angle tenderness. Her heart rate was 96 beats per minute and blood pressure was 155/90 mmHg. The findings of laboratory tests, which were complete blood count, liver and renal function tests, and urine analysis, were within normal range and they did not help us find the etiology of her right flank pain. Abdominal screening with computed tomography (CT) revealed a mass on her right kidney, which was considered a primary renal cell carcinoma and she underwent a right nephrectomy. Unexpectedly, PTC metastasis was diagnosed from demonstrative histopathological findings, such as positive immunoperoxidase staining for thyroglobulin (Tg). After further examinations of her thyroid and neck with ultrasonography (USG), a total thyroidectomy was performed. Pathological examination of thyroid tissue revealed a 5 cm tumor with capsular invasion and a strong positive immunoperoxidase staining of cytokeratin-19, HBME-1, and galectin-3. She was diagnosed as having metastatic PTC. Orally administered levothyroxine 75 mcg daily was initiated in addition to the metformin 1000 mg twice daily and amlodipine 10 mg daily treatments she received prior to PTC diagnosis. Postoperative serum Tg was above 300 ng/ml and anti-Tg was negative. Afterward, she was screened with unenhanced thoracic CT and skeletal scintigraphy. They revealed bilateral multiple nodules in her lungs and bone metastasis on T10 vertebra and right sacroiliac joint. Initially, 30 Gy radiotherapy was implemented to her T9–10 vertebrae for 12 days. We also started treating her with L-thyroxine to keep her thyrotropin (TSH) level below 0.1 mIU/L. After 2 months, she was treated with 200 mCi RAI for ablation. A RAI whole body scan (WBS) showed extensive RAI uptake in lungs and bones. A second 200 mCi RAI was applied 8 months after the first treatment. A post-ablative WBS showed progression. Serum Tg was still above 300 ng/ml and 200 mCi RAI administration was applied for the third time. A WBS was still displaying high radioactive activities in multiple areas of her body. Because of the existence of increased uptake, we planned a fourth RAI treatment but our patient was lost to follow-up for 2 years.

**Fig. 1:**
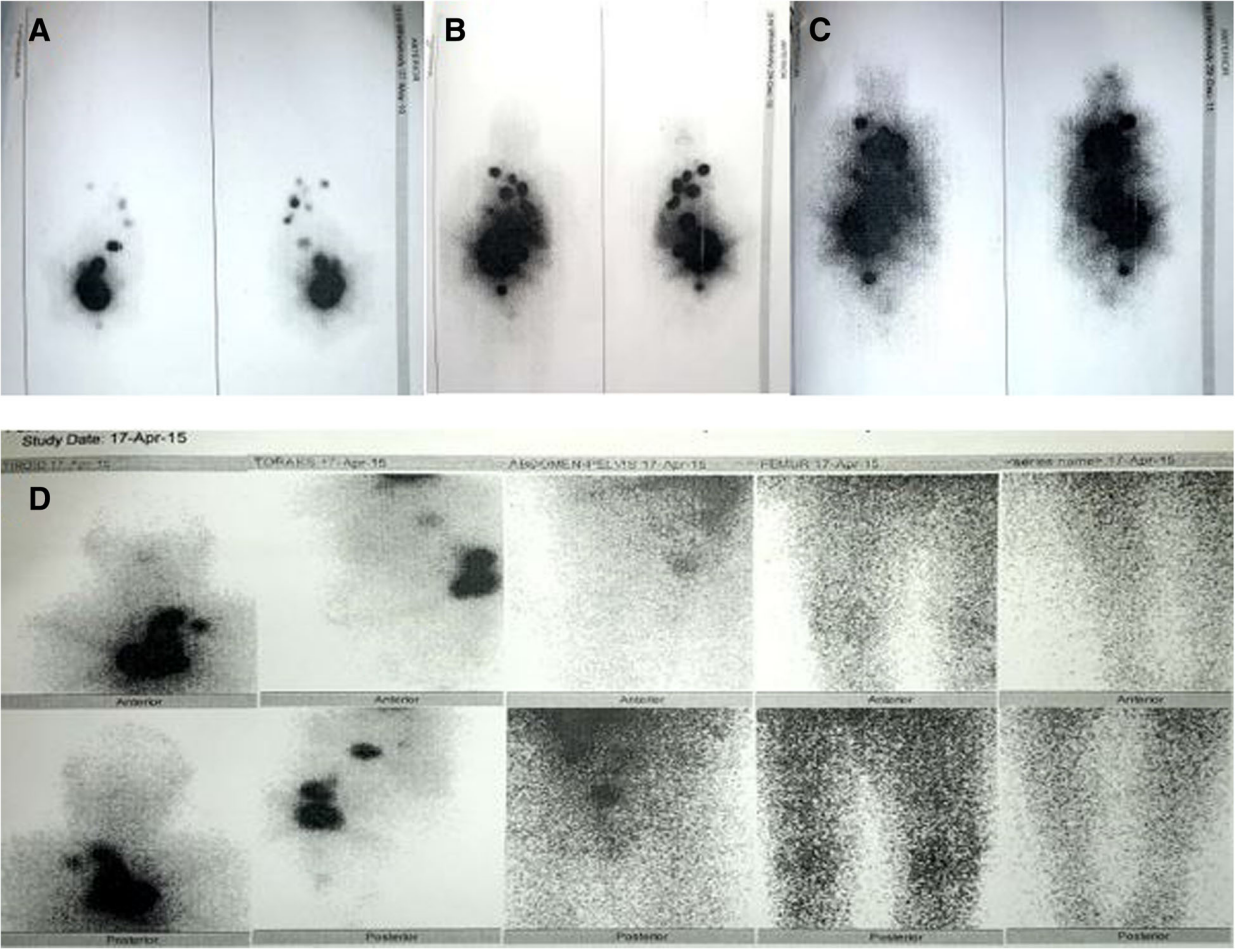
Whole body scan images after first (**a**), second (**b**), third (**c**), and fourth (**d**) radioactive iodine treatment

**Fig. 2:**
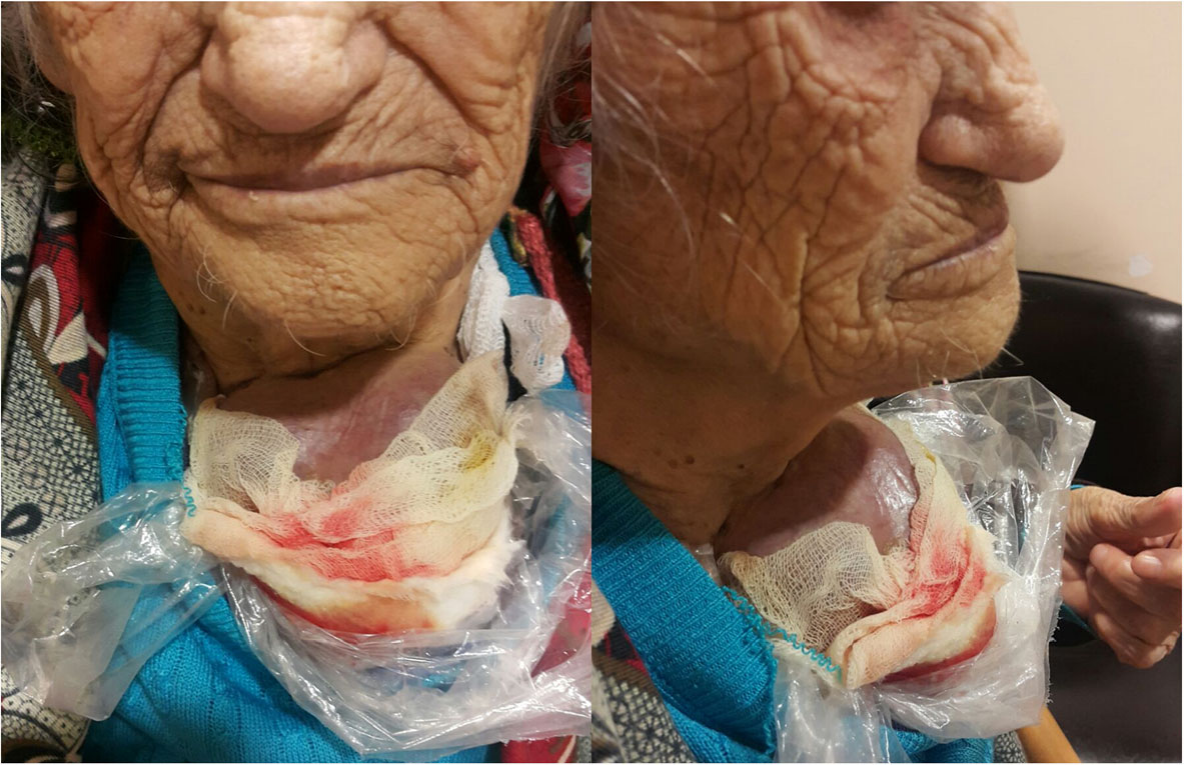
Huge hemorrhagic draining cervical mass

**Fig. 3:**
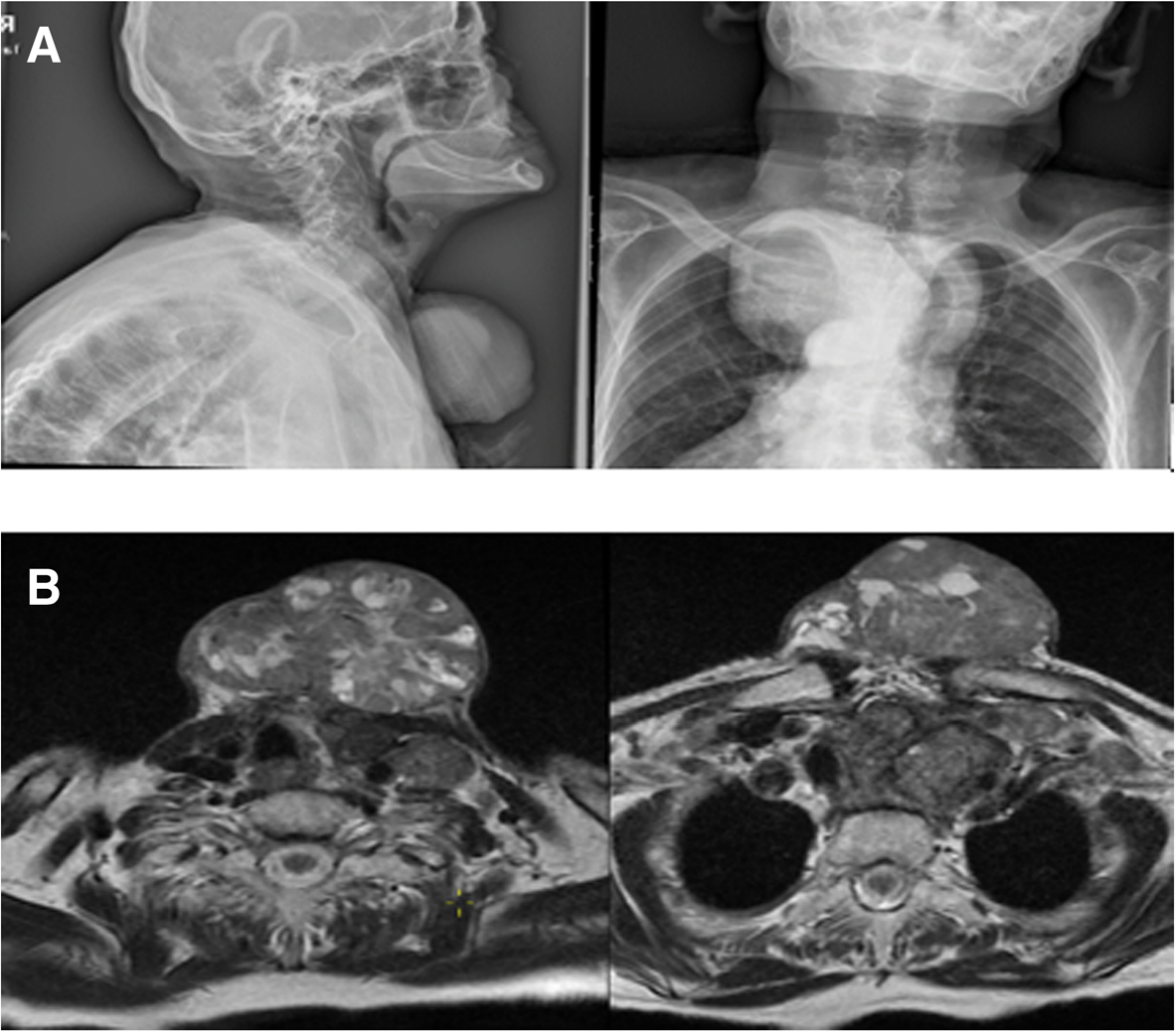
Screening images of the cervical mass, displayed by lateral anteroposterior X-ray (**a**) and cervical magnetic resonance imaging (**b**)

When she presented again in 2015, her serum Tg was above 300 ng/ml again and a fourth 200 mCi RAI WBS of our patient was done. Unexpectedly, the WBS revealed diminished RAI uptake compared with the previous ones (Fig. 1). However, a neck USG showed two solid thyroid nodules at the previous thyroid area and bilateral lung metastases were identified by thoracic CT. For the next 3 years, she was lost to follow-up, again. Finally, in February 2018, she was referred to our clinic and presented with a huge hemorrhagic draining cervical mass (Fig. 2). Of interest, besides this finding, she did not have any other complaints other than a little dyspnea when lying down. Summing up all previous RAI treatments, cumulative 800 mCi RAI was given to her in the past 10 years; however, a physical examination and screening findings were not yet promising at the last follow-up (Fig. 3). Eventually, considering her elderly age, harboring multiple metastases, and the absence of severe complaints, we planned radiotherapy to the giant mass on her neck. After applying radiotherapy, she was lost to follow-up again and at the end of the year her sons reported that she was dead.

## Discussion

The present case is an 85-year-old woman with no previous history of thyroid disease who referred to our hospital’s urology clinic with the only complaint of right flank pain. Since initial symptoms with renal metastasis are so rare, thyroidal disease is not usually considered in the differential diagnoses. Consequently, our patient was misdiagnosed and underwent radical nephrectomy to cure renal cell carcinoma 10 years ago. However, subsequently, some histopathological examinations and some cytological findings revealed that the tumor originated from PTC. Therefore, it would be challenging to diagnose the metastatic tumor before nephrectomy. By reporting this case, we wanted to suggest considering a primary renal mass as a possible metastasis from the thyroid and sharing this differential diagnosis with colleagues working at a pathology department might be beneficial for diagnosis.

Distant metastasis of DTC is infrequent and the metastasis rate of PTC is lower than that of FTC. While lung and bone metastases are more frequent, renal metastasis of DTC is uncommon. In the English language literature, there are only approximately 30 cases of renal metastasis of DTC and most of them are FTCs. In a retrospective analysis of 1038 patients with DTC, 44 patients presented initially with distant metastases (4%) and only 19 of them were with PTC (2.3%) [4]. Similarly, Benbassat *et al.* reported that 44 patients had distant metastases out of 660 patients with DTC (6.7%), with a prevalence of 4.8% for PTC and 21% for FTC [1]. The authors reported that affected sites were lungs, bone, brain, and uterus but not kidneys. Considering the presence of only a few cases with PTC introduced as primary renal tumors [5, 6], we thought this case worthy to share.

Some studies revealed the low survival rates of patients with metastatic DTC. Degroot *et al.* demonstrated a 47-fold increased risk of death in patients with PTC with distant metastases compared with those with localized PTC [7]. In different retrospective analyses the 10-year survival rates of DTC with distant metastases were variable. Benbassat *et al.* investigated 660 patients with DTC and found 77% had 10-year survival rate of metastatic disease, whereas Elisei *et al*. showed that the rate for 4187 patients with DTC was below 50% [1, 8]. The median disease-specific survival rate of patients with brain metastasis was 16.7 months in an analysis including 47 cases [9]; however, the survival rate of patients with DTC with renal metastasis is not demonstrated yet because of its rarity. In our case, our patient is an 85-year-old woman and the renal metastasis of PTC was diagnosed 10 years ago. On checking our patient at first sight, it could be estimated that the prognosis would be poor in terms of multiple lung, bone, and renal metastases; however, she was still alive without any major complaint besides a neck mass.

Before discussing the unexpected long-term survival of the present case, the main treatment applied to our patient should be mentioned. A total of 800 mCi RAI was given and, taking into consideration the last WBS results, it can be stated that the metastatic malignancy of our patient was quite sensitive to RAI. On the other hand, the presence of both thyroidal and extrathyroidal huge masses indicated that the cumulative dose was not sufficient. It can be pointed out that 800 mCi RAI was adequate to keep her survival long, but not enough for a disease-free survival. This situation could be explained by a study in which Sherman identified decreased or even the absence of iodine sensitivity of distant metastases of DTC [10]. For this reason, we decided to use radiotherapy locally to her neck region considering both her age and multiple metastases at the same time.

## Conclusion

Distant metastatic disease presenting as a primary renal tumor in PTC is unusual. Various treatment modalities such as surgical, nuclear, or even medical for extensively metastatic PTC can be used per se or in combination according to the characteristics of patients. Even though distant metastatic disease causes significantly poor prognosis, long-term survival can still be promising. In addition, in relation to primary renal tumors, possible distant metastases of other organs need to be kept in mind for differential diagnosis.
